# Comparative Evaluation of Corrosion Resistance of AISI 316L and Ti6Al4V Dental Materials Under Simulated Inflammatory Conditions

**DOI:** 10.3390/ma18102243

**Published:** 2025-05-12

**Authors:** Mojca Slemnik

**Affiliations:** Faculty of Chemistry and Chemical Engineering, University of Maribor, Smetanova Ulica 17, SI-2000 Maribor, Slovenia; mojca.slemnik@um.si

**Keywords:** corrosion, EIS, inflammatory conditions, titanium, stainless steel, dental materials

## Abstract

Titanium and its alloys, as well as stainless steel, are commonly used materials for implants in the human body due to their excellent biocompatibility, corrosion resistance, and mechanical properties. However, the long-term performance of these materials in the oral cavity can be affected by the complex oral environment, including the ingestion of food, beverages, and oral hygiene products, leading to the presence of various ions, pH fluctuations, and inflammatory processes. In this study, the corrosion properties of two biocompatible materials, Ti6Al4V and AISI 316L stainless steel, are investigated under varying oral inflammatory conditions. Using potentiodynamic polarization, electrochemical impedance spectroscopy (EIS), SEM, and EDS analysis, the corrosion behaviour of both materials was analysed in environments simulating mild and severe inflammation. Results indicate that Ti6Al4V exhibits superior corrosion resistance at low H_2_O_2_ concentrations mimicking mild inflammation, with significantly lower corrosion rates compared to AISI 316L. However, at higher H_2_O_2_ concentrations, which correspond to severe inflammation, AISI 316L shows better resistance despite its susceptibility to pitting corrosion. Both alloys show reduced passivation after 72 h, with corrosion products accumulating on the surface after 96 h, contributing to repassivation. These results emphasise the need for individualized material selection in dental applications based on a patient’s susceptibility to oral inflammation.

## 1. Introduction

Titanium and its alloys, such as Ti6Al4V, are known for their superior biocompatibility and osseointegrative properties, making them a popular choice for dental implants for years [1]. However, the potential release of titanium ions and particles due to corrosion processes can lead to adverse tissue reactions, known as peri-implantitis [2,3,4,5,6]. On the other hand, stainless steel such as AISI 316L, used in orthopaedics, dentistry, and orthodontics, has demonstrated good corrosion resistance also in the oral environment, but concerns have been raised about the long-term release of metal ions, such as chromium and nickel, and their potential cytotoxicity [7].

Titanium has the amazing ability to form a stable oxide layer of TiO_2_ with oxygen, which further protects the metal surface from corrosion or greatly slows it down. Most new research is focused on improving titanium and its alloys through various processes that reduce its corrosiveness and increase its biocompatibility in medical applications. The corrosion rate can be reduced by increasing the thickness of the protective layer of TiO_2_ on the metal surface by various methods, such as: selective laser melting, which induces changes in the microstructure of the alloy [8,9], the anodization [10], sol-gel method [11], sputtering, and evaporation [12], or thermal oxidation [13]. Electrolytic oxidation [14] and surface modifications, which generate a mixed oxide layer resembling the extracellular matrix of two TiO_2_ layers [15], improve the bone osseointegration. This can also be improved by the development of innovative materials such as Ti-Zr [16], Ti-Zr-Mo-Mn [17], Ti-Nb [18], Ti-Zr-Nb [19], Ti-Ag [20], Nb [21], Ti in trabecular form [22], Ta in trabecular form [23,24], and others.

AISI 316L low-carbon stainless steel is the most commonly used material for orthodontic brackets and crowns, offering high tensile strength and good corrosion resistance [25]. It shows excellent biocompatibility and has been studied in various simulated body fluids, such as artificial saliva, artificial saliva with fluoride ions [26], mouthwash solutions [16], simulated oral environment via extracellular electron transfer and acid metabolites of subgingival microbiota [27], microbiologically influenced corrosion [28], borovine serum [29], lactic and phosphoric acid in artificial saliva [30], or by adding some effective plant extracts to mouth hygiene products [31].

AISI 316L steel has excellent biocompatibility properties and forms a protective layer on its surface consisting mainly of Cr oxides, mostly Cr_2_O_3_, which prevents and slows down further corrosion [16,25,26]. The oxides of the other elements are low or negligible compared to the bulk concentration. The main problem with the AISI 316L alloy is undoubtedly its tendency to pitting corrosion, which increases with increasing levels of Cr, Ni, and Mo [16,26]. Pitting corrosion resistance can also be predicted using neutron scattering in combination with scanning electron and atomic force microscopy by determining the pitting corrosion equivalent number (PREN) and precise composition determination [32]. Further problems can arise from bacterial infections, which is why several studies are being conducted on the addition of copper or silver to AISI 316L [33,34,35,36]. Microbiologically-induced intergranular corrosion and localized pitting of AISI 316L stainless steel dental material can also be accompanied by the evolution of volatile sulphur compounds within this anaerobic culture [28].

Scientific studies on dental materials are often conducted in model solutions containing fluoride ions, which are present in oral hygiene products, or in sodium chloride (NaCl) [37,38,39,40,41,42] solutions that simulate exposure to salt, which contains highly corrosive chloride ions. However, within the oral cavity, inflammation of the surrounding tissues frequently occurs, significantly influencing the corrosion behaviour of dental alloys. To address these factors, this study aims to compare the corrosion behaviour of AISI 316L stainless steel and Ti6Al4V dental implant materials, as well as other dental accessories, in artificial saliva under simulated inflammatory conditions. Inflammation-related conditions are mimicked using hydrogen peroxide (H_2_O_2_) and lactic acid. Namely, during inflammation resulting from abscesses or some surgical trauma, hydrogen peroxide is released by both bacteria and leucocytes [23,30,43,44,45,46], which can act as a driving force for the enhanced dissolution and regrowth of the passive layer on the metal surface.

In this work, a direct comparison is made between the two most used biocompatible materials, Ti6Al4V and AISI 316L, used in this case as dental materials in the oral cavity, using rapid electrochemical methods to analyse their corrosion resistance. The immersion of the samples over a period of 24, 48, 72, and 96 h and different H_2_O_2_ concentrations represents the progressive intensity of the inflammation and the effects on the passivation of the materials. Concentrations of 0.1% H_2_O_2_ and 0.5% H_2_O_2_ mimic mild inflammation, while 10% H_2_O_2_ mimics severe and acute inflammation, respectively.

The study will employ various scientific techniques, including electrochemical impedance spectroscopy, scanning electron microscopy, and potentiodynamic electrochemical measurements, to evaluate the corrosion resistance and corrosion rate of both materials.

The findings of this research will contribute to the understanding of the long-term performance and suitability of these dental implant materials in the challenging oral environment, ultimately aiding in the selection of the most appropriate material for dental implant applications.

## 2. Materials and Methods

The samples AISI 316L (mass %: C ≤ 0.03; Mn ≤ 2.00; P ≤ 0.045; S ≤ 0.03; Si ≤ 0.75; Cr 16–18; Ni 10–14; Mo 2.00–3.00; N ≤ 0.10; Fe balanced; Goodfellow, Cambridge Ltd., Huntingdon, UK) and Ti6Al4V (mass %: Ti 90; Al 6; V 4; C < 0.10; O < 0.20; N < 0.50; H < 0.0125; Fe < 0.3; Goodfellow, Cambridge Ltd., UK), with a diameter of 10 mm and a thickness of 3 mm, were cut into shape of discs. Mechanical polishing was conducted using 400–1200 abrasive papers, followed by a treatment with diamond pastes to achieve a mirror-like quality. Once the samples were well ground and polished, they were degreased in ethanol and cleaned in an ultrasonic bath for 10 min. Finally, the samples were dried with compressed air and weighed.

To the modified Fusuyama artificial saliva (KCL—0.4 g/L, NaCl—0.4 g/L; CaCl_2_H_2_O—0.906 g/L Na_2_H_2_PO_4_ × 2H_2_O—0.96 g/L, Urea—1 g/L), 0.1% H_2_O_2_, 5.0% H_2_O_2_, and 10.0% H_2_O_2_ were added, respectively. Lactic acid was added to all solutions to achieve a pH value of 4.5, which corresponds to the pH value of dental plaque in the oral cavity. The measurements were performed after 24, 48, 72, and 96 h after passivation of the samples at 37 °C.

The electrochemical measurements were conducted in a standard three-electrode cell with the sample as the working electrode, platinum as the counter electrode and the reference SCE. The cell was thermostated at 37 °C and filled with 300 mL of the prepared solution. The measurements were conducted using a Solartron 1287 electrochemical interface (Solartron Analytical, Farnborough, UK) and a Solartron 1250 frequency response analyser. The impedance curves were recorded at the OCP in the frequency range from 60 kHz to 1 mHz. The amplitude of the excitation voltage was set to 10 mV. EIS measurements were performed 20 min after polarization, and data were acquired and processed using ZPlot and ZView software, developed by Scribner Associates, Inc. (Southern Pines, NC, USA) [47].

Potentiodynamic curves were plotted within the potential range of −0.6 V to 1.0 V vs. SCE at a sampling rate of 1 mVs^−1^. Data were recorded using CorrWare and processed using CorrView software, both developed by Scribner Associates, Inc. [47]. All experiments were conducted in three parallel experiments with a deviation of up to 3%.

The surface morphology of the passive layers was analysed with a Scanning Electron Microscope (Sirion 400 NC, Eindhoven, The Netherlands), and the microchemical analysis was performed with the EDS (Energy Dispersive Spectroscopy) INCA 350.

## 3. Results and Discussion

### 3.1. Surface Analysis

**Figure 1:**
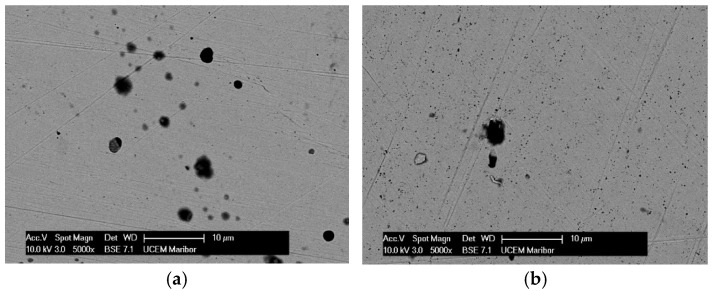
SEM images for AISI 316L: (**a**) Artificial Saliva, (**b**) Artificial saliva with 10% H_2_O_2,_ both after 96 h of immersion.

**Figure 2:**
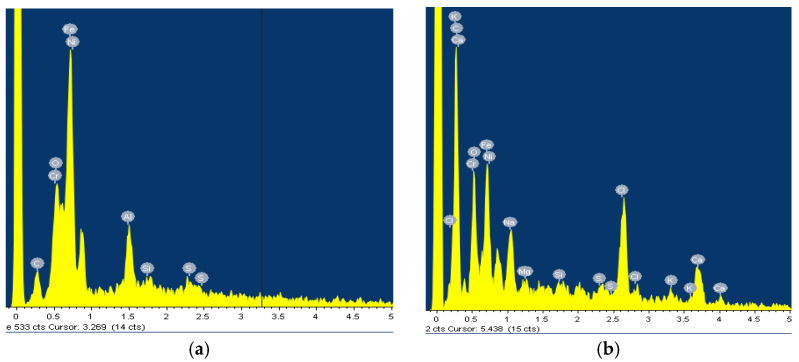
EDS for AISI 316L: (**a**) Artificial Saliva, (**b**) Artificial saliva with 10% H_2_O_2_, both after 96 h of immersion.

**Figure 3:**
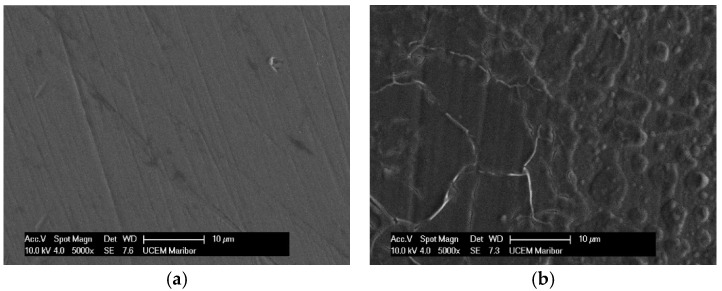
SEM images for Ti6Al4V: (**a**) Artificial Saliva, (**b**) Artificial saliva with 10% H_2_O_2_, both after 96 h of immersion.

**Figure 4:**
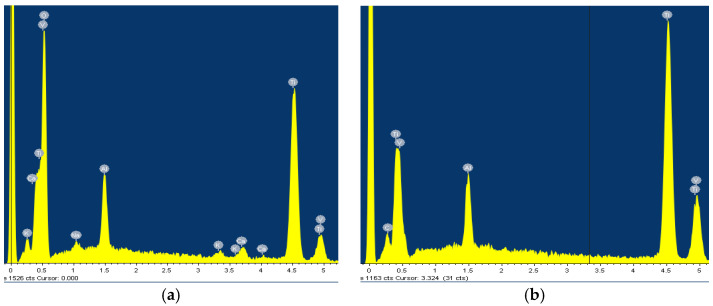
EDS for Ti6Al4V: (**a**) Artificial Saliva, (**b**) Artificial saliva with 10% H_2_O_2_, after 96 h of immersion.

After the samples had been thermostated in pure artificial saliva and artificial saliva with different concentrations of H_2_O_2_, they were passivated, i.e., coated with a protective layer that further reduces corrosion processes. The morphology of the passive layers and the composition of their elements were analysed using scanning electron microscopy (SEM) and EDS. Figure 1 and Figure 2 show the morphology of the treated samples e and EDS results for AISI 316L, while Figure 3 and Figure 4 show SEM and the EDS results for Ti6Al4V. Images of samples immersed in artificial saliva and in artificial saliva with 10% H_2_O_2_ are shown, as these represent the extreme experimental conditions.

The surface of the AISI 316L appears smooth with some dark spots identified as Cr, Ni, O, and Fe by the EDS method, but the largest spot had an increased carbon content (Figure 1b), which could indicate pitting corrosion with carbide formation. After the steel was immersed in H_2_O_2_, the surface changed slightly. The highest percentage of oxygen is 26.08 wt.% and chromium 29.95 wt.% at the selected test site, indicating a passive layer of chromium oxide.

While the lamellar microstructure of the Ti6Al4V surface can be clearly recognized in Figure 3a, the phases α (dark grains) and β (bright contrast—on black scattered electron images) can be clearly distinguished on the surface after passivation in 10% of H_2_O_2_ (Figure 3b), as has also been reported by other authors [4,14,21,48]. The phase β is located at α grain boundaries and triple junctions. At the start, in pure artificial saliva, only the high content of titanium is detected by the EDS method: 90.03 wt.%, with the rest being vanadium and aluminium (Figure 4a). After 96 h of immersion in 10% H_2_O_2_, a high oxygen content was found in the dark grains: 35.55 wt.%, 56.54 wt.% titanium, and the remainder being vanadium and aluminium (Figure 4b), indicating the formation of TiO_2_, while the β phase (bright lines) contains more titanium −75.35 wt.% and has a lower aluminium/vanadium ratio: 4.37/2.77 wt.%. Phase α corresponds to the oxide layer and represents a good passivation, while phase β represents a more open structure with pores in which the oxide layer is also present but thinner [21].

### 3.2. Potentiodynamic Measurements

**Figure 5:**
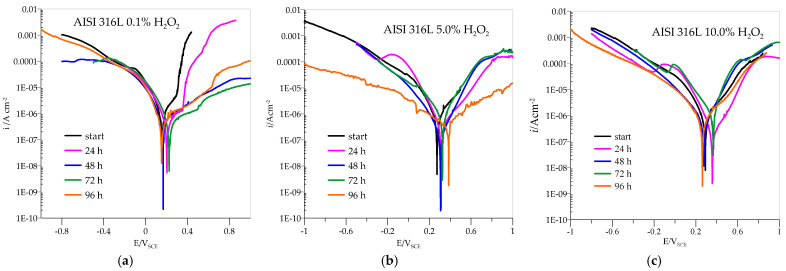
Polarization curves for AISI 316L in different concentrations of H_2_O_2_: (**a**) 0.1%, (**b**) 5.0%, (**c**) 10.0%.

The anodic polarisation curves of AISI 316L are shown in Figure 5, and the parameter values from the curves are listed in Table 1, with *i*_corr_ standing for the corrosion current density at potential *E*_corr_, which is determined by Tafel extrapolation. The value of *i*_corr_ can then be used in specific mathematical equations to calculate the corrosion rate; *i*_pas_ stands for the current density at *E*_pas_, which occurs at the beginning of the passivation region. The samples were immersed in pure artificial saliva and in artificial saliva with H_2_O_2_ at different concentrations: 0.1, 5.0, and 10.0%. All samples showed immediate passivation. At 0.1% H_2_O_2_, the corrosion potential is slightly shifted in the positive direction. The corrosion current decreases with the time the samples are immersed in the medium, which means that the passivity becomes more efficient with time. If the concentration of H_2_O_2_ is increased to 5.0%, the potential values shift towards positive values, and the corrosion currents are also reduced. This indicates that the increased concentration in this case further accelerates the passivation of AISI 316L.

At a concentration of 10.0% H_2_O_2_, the potential values do not change significantly, but the corrosion currents increase considerably so that passivation is less effective. At both concentrations, 5.0 and 10.0% H_2_O_2_, a peak value of the corrosion current is observed after 72 h of immersion, which means that the material corrodes the most during this time, but the corrosion current decreases again after 96 h, indicating a repassivation process.

**Figure 6:**
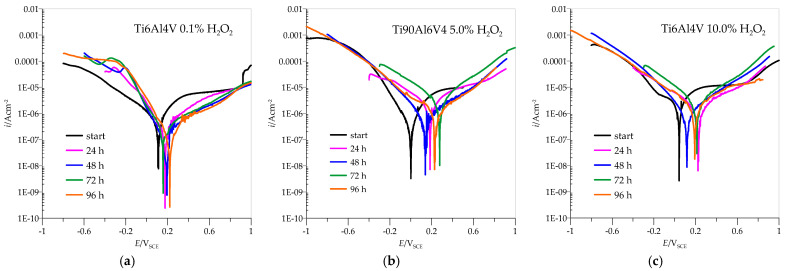
Polarization curves for Ti6Al4V in different concentrations of H_2_O_2_: (**a**) 0.1%, (**b**) 5.0%, (**c**) 10.0%.

Potentiodynamic curves for Ti6Al4V presented in Figure 6 showed that, in the anodic region, no well-defined Tafel slopes were observed, suggesting that the samples exhibit passive behaviour. In such a case, the corrosion rate is defined by the passive current density [45]. The passivation current densities were determined from the plateau areas of the anodic curves shown in Figure 6 and are listed in Table 2.

At a concentration of 0.1% H_2_O_2_, the corrosion currents are quite low, especially compared to AISI 316L, and the potentials move in positive directions with the immersion time. The highest corrosion current is observed at the beginning, and the lowest after 96 h of immersion, indicating excellent passivation that improves with time. However, already when the concentration of H_2_O_2_ is increased to 5.0%, the picture regarding AISI 316L changes drastically. The corrosion currents are higher in all cases, and the potential values do not move towards higher positive values. Again, the corrosion current is highest at the beginning and after 72 h of immersion but decreases after 96 h, indicating the good possibility of repassivation. When the concentration of H_2_O_2_ increases to 10.0%, the corrosion currents increase significantly and are consistently above the values for AISI 316L. The maximum value is again reached after 72 h of immersion, but with a possibility of repassivation if the immersion time is at least 96 h.

### 3.3. EIS Measurements

The fitting procedure was conducted using equivalent circuits known as the modified Randle’s circuit with RC elements in series with the solution resistance. *R*_s_ and *R*_pl_ stand for electrolyte (solution) resistance and the resistance of the oxide layer, respectively. Element *C* represents the capacitance that exists at the interface between an electrode and the surrounding electrolyte. Instead of *C*, the element CPE is usually used for capacitance calculation in connection with the passive layer, which describes the non-ideal behaviour of the capacitor. It provides better agreement between experimental and theoretical data, especially when the experimentally obtained impedance curves are not ideal semi-circles, which is a criterion for the inhomogeneity and irregularities of corrosive systems. The impedance of a constant phase element is defined as [49,50,51]:

*Z*_CPE_ = [*Q* (j*ω*)*^n^*]^−1^ (1)

where *Q* is the frequency-dependent element (constant phase element, CPE) and is a combination of properties related to both the surface area and the electroactive species. The exponent *n* is related to the slope of log *Z* versus log *f* in the Bode diagram, i.e., to the phase angle *θ* by the relation *n* = 2*θ*/π; j = (−1)^0.5^. *ω* is the angular frequency. For *n* = 1, the *Q* is reduced to a capacitor with capacitance *C*, and for *n* = 0, to a pure resistor. *n* = 0.5 provides the Warburg impedance that occurs when a charge carrier diffuses through a material. The parameter *Q* (s^n^ Ω^−1^ cm^−2^) can be converted into capacitance *C* (F cm^−2^) when *n* < 1, which is particularly important when using experimental data to quantify system parameters such as thickness or dielectric constant.

**Figure 7:**
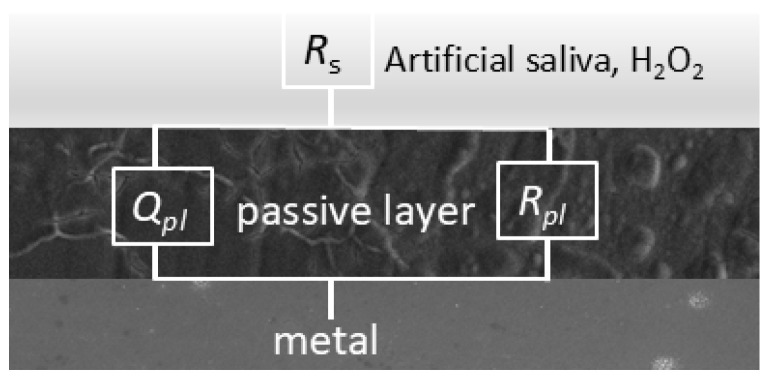
Equivalent circuit used for modelling the EIS results.

The equivalent circuit for the simple passivation system shown in Figure 7 was used for fitting the data.

**Figure 8:**
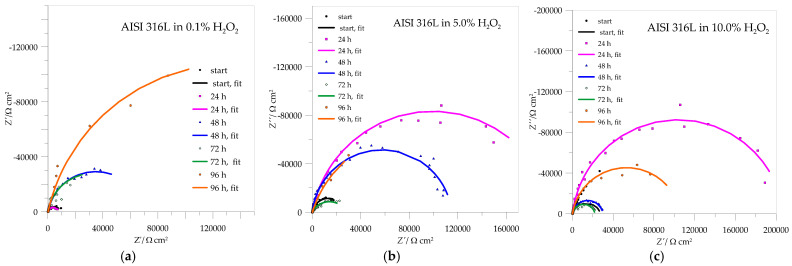
Impedance spectra for AISI 316L in different concentrations of H_2_O_2_: (**a**) 0.1%, (**b**) 5.0%, (**c**) 10.0%.

Figure 8 shows impedance data for AISI 316L. The Nyquist curves are typical for high-impedance systems that do not reach a low frequency limit and have only a partial semicircle, indicating an excellent passivation process. The parameter values from the curves are summarized in Table 3.

Figure 8a clearly shows that the resistances of the resulting passive layers increase with passivation time and reach a maximum value after 96 h of immersion in 0.1% H_2_O_2_, which represents the best passivation in this case. In the case of 5.0% H_2_O_2_, the *R*_pl_ still increases after 24 h of immersion but decreases after 48 and even more after 72 h of immersion. After 96 h, there is a visible increase in the resistance of the protective layer, which represents a repassivation in which there is an obvious increase in the deposition of corrosion products that further protects the metal surface. After increasing the H_2_O_2_ concentration to 10.0%, all *R*_pl_ values decreased, with good passivation observed after 24 h of immersion and slight repassivation again after 96 h.

**Figure 9:**
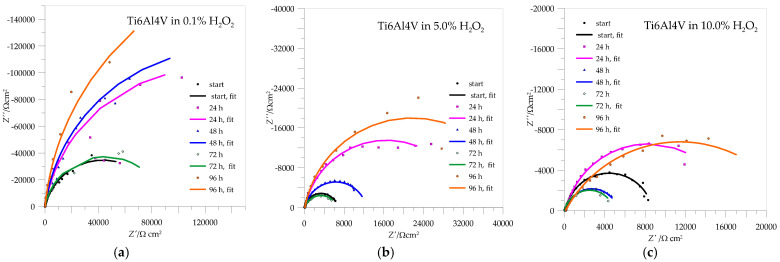
Impedance spectra for Ti6Al4V in different concentrations of H_2_O_2_: (**a**) 0.1%, (**b**) 5.0%, (**c**) 10.0%.

The impedance spectra for Ti6Al4V are shown in Figure 9, and the calculated values of the parameters from the curves are collected in Table 4. In these cases, the Nyquist curves are also typical of passive systems in which only part of a semicircle is visible.

As with AISI 316L, the resistance of the passive layer of Ti6Al4V in 0.1% H_2_O_2_, which represents a deposit of compact TiO_2_, also increases with the immersion time, with the lowest value being reached after 72 h of passivation and the highest after 96 h—the repassivation of the protective layer can also be confirmed here. When the concentrations of immersion media increase to 5.0 and 10.0% H_2_O_2_, the resistances of the passive layers drop drastically and offer less corrosion protection, but in both cases, there is a minimal possibility of repassivation after 96 h.

**Figure 10:**
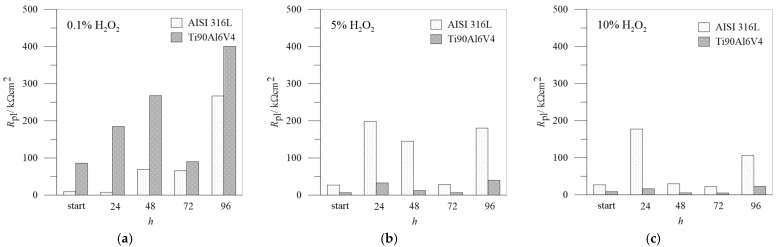
*R*_pl_ values from impedance spectra: comparison between AISI 316L and Ti6Al4V in different concentrations of H_2_O_2_: (**a**) 0.1%, (**b**) 5.0%, (**c**) 10.0%.

For a better comparison between AISI 316L and Ti6Al4, the data of the *R*_pl_ of the passive layers as a function of the exposure time for different H_2_O_2_ concentrations are summarized in Figure 10.

### 3.4. Corrosion Rate

**Figure 11:**
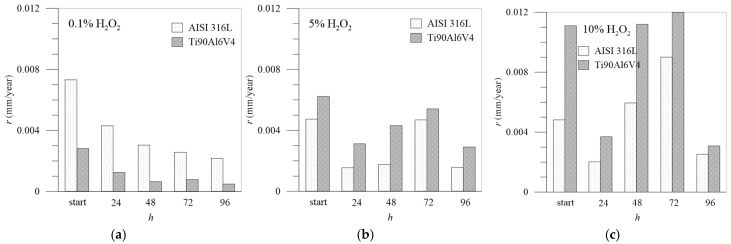
Corrosion rates in mm/year: comparison between AISI 316L and Ti6Al4V in different concentrations of H_2_O_2_: (**a**) 0.1%, (**b**) 5.0%, (**c**) 10.0%.

The resistances of the passive layer *R*_pl_ agree well with the calculated corrosion rates shown in Figure 11, which shows the corrosion rate between the two tested materials. The corrosion rates were calculated from the potentiodynamic curves at different time intervals based on the Tafel extrapolation from the polarization curves, representing the logarithmic current density as a function of overpotential *η* [52]. Firstly, Tafel constants and corrosion current density, *i*_corr_, are determined, and then corrosion rate, *r*_corr_, is calculated from the equation via Faraday’s law in terms of penetration rate (mm/year) [53]:

(2) $$r_{corr}=\frac{M\cdot I}{n\cdot \rho \cdot F\cdot A}$$

*M* stands for the molar mass and *n* is the number of electrons in the corrosion reaction. *M/n* are summarised in the ASTM G102 standard for various materials [53]. *I* is the corrosion current, and *A* is the exposed area. *I*/*A* stands for *i*_corr_ calculated for *A* = 1 cm^2^. F is a Faraday constant, and ρ stands for the density of the material.

The results are summarized in Table 5:

When the concentration of the aggressive medium is low (0.1% H_2_O_2_), the corrosion rates are also low overall. The corrosion rates of Ti6Al4V are lower than those of AISI 316L and decrease with the passivation time, with the lowest value being reached after 96 h of exposure to the medium. This all points to the extremely good protective function of the passivation layer made of TiO_2_, which becomes increasingly compact over time and is effectively protective against further corrosion.

At higher concentrations of H_2_O_2_, the picture changes considerably: Ti6Al4V is initially slightly better protective than AISI 316L, but as the immersion time increases, its performance is reduced. It is assumed that the chromium forms insoluble Cr_2_O_3_, creating a continuous network of Cr–O–Cr–O that prevents the dissolution of iron [26]. At 5.0% H_2_O_2_, Ti6Al4V is the least protected after 72 h, but after 96 h, a sufficient protective layer has apparently been deposited on the surface to reduce the further corrosion rate. It can also be assumed that at shorter time intervals, the formation of sub-oxides TiO and Ti_2_O_3_ occurs [40], which, after 72 h, completely transform into TiO_2_, which forms a high protective layer on the surface so that the corrosion resistance increases after 96 h of passivation. In artificial saliva with 10.0% H_2_O_2_, Ti6Al4V reaches its maximum corrosion rate at the first moment of immersion in the medium, then passivates relatively well, but the corrosion rate increases again after 48–72 h. The corrosion rates of stainless steel in artificial saliva with 5.0% H_2_O_2_ indicate such passivation, which provides better protection of the material’s surface and a relatively low corrosion rate compared to Ti6Al4V. The corrosion rates of AISI 316L increased sharply in artificial saliva with 10.0% H_2_O_2_ and became highest after 72 h of immersion but decreased significantly again after 96 h. Finally, if we compare the corrosion rates of the two test materials, the corrosion rate of AISI 316L is, on average, around 30% lower than the corrosion rate for Ti6Al4V in 5.0% H_2_O_2_ and almost 50% lower in the case of 10.0% H_2_O_2_.

## 4. Conclusions

The essence of this study is to compare the corrosion properties of the two biocompatible materials, which can be compared using rapid electrochemical methods, and, above all, determine their corrosion severity to allow individualised treatment of patients depending on their tendency to oral inflammation. The measured values and the consistent results with potentiodynamic and EIS methods clearly indicate good passivation of both test materials, with a protective layer of TiO_2_ being applied to Ti6Al4V and chromium oxides to AISI 316L. At low concentrations of H_2_O_2_, which corresponds to the mild inflammatory conditions in the oral cavity, Ti6Al4V performs better, as its corrosion rates in this case are very low, between 0.003 and 0.0005, while these values for AISI 316L are between 0.007 and 0.002 mm/year in terms of penetration. Interestingly, the situation changes at higher H_2_O_2_ concentrations, presenting an extensive inflammation: as expected, all corrosion rates increase, and the resistance of the protective layers decreases accordingly, but AISI 316L reacts surprisingly better than the titanium alloy. The problem that arises here is the tendency of AISI 316L to pitting corrosion. What both alloys have in common is that they exhibit the worst passivation after 72 h, which also means that most corrosion products are brought to the surface, while after 96 h, these products are obviously deposited on the surface and, thus, abruptly reduce the corrosion rate or contribute to the repassivation of the protective layer.

Considering this study, it is, therefore, important to decide which material should be used in patients who may be more susceptible to oral inflammation, but the decision as to which material is more suitable for a particular patient is still left to the medical staff.

## Figures and Tables

**Table 1:** Parameter values from potentiodynamic curves for AISI 316L.

	0.1% H_2_O_2_				5.0% H_2_O_2_				10.0% H_2_O_2_			
*h*	*i*_corr_ (A/cm^2^)	*E*_corr_ (V_SCE_)	*i*_pas_ (A/cm^2^)	*E*_pas_ (V_SCE_)	*i*_corr_ (A/cm^2^)	*E*_corr_ (V_SCE_)	*i*_pas_ (A/cm^2^)	*E*_pas_ (V_SCE_)	*i*_corr_ (A/cm^2^)	*E*_corr_ (V_SCE_)	*i*_pas_ (A/cm^2^)	*E*_pas_ (V_SCE_)
0	6.93·10^−7^	0.1573	4.69·10^−8^	0.159	4.54·10^−7^	0.2738	7.52·10^−7^	0.309	4.62·10^−7^	0.2908	2.81·10^−8^	0.291
24	4.12·10^−7^	0.2032	5.05·10^−8^	0.205	1.48·10^−7^	0.3120	7.8·10^−9^	0.314	1.93·10^−7^	0.3580	9.05·10^−9^	0.359
48	2.92·10^−7^	0.1659	0.159·10^−7^	0.168	1.69·10^−7^	0.3074	1.23·10^−8^	0.308	5.78·10^−7^	0.2770	5.55·10^−8^	0.280
72	2.47·10^−7^	0.2252	1.097·10^−7^	0.230	4.50·10^−7^	0.3262	4.78·10^−9^	0.320	8.62·10^−7^	0.3671	8.98·10^−8^	0.367
96	2.08·10^−7^	0.1510	3.59·10^−8^	0.153	1.51·10^−7^	0.3840	5.44·10^−8^	0.386	4.61·10^−7^	0.2626	2.19·10^−8^	0.263

**Table 2:** Parameter values from potentiodynamic curves for Ti6Al4V.

	0.1% H_2_O_2_				5.0% H_2_O_2_				10.0% H_2_O_2_			
*h*	*i*_corr_ (A/cm^2^)	*E*_corr_ (V_SCE_)	*i*_pas_ (A/cm^2^)	*E*_pas_ (V_SCE_)	*i*_corr_ (A/cm^2^)	*E*_corr_ (V_SCE_)	*i*_pas_ (A/cm^2^)	*E*_pas_ (V_SCE_)	*i*_corr_ (A/cm^2^)	*E*_corr_ (V_SCE_)	*i*_pas_ (A/cm^2^)	*E*_pas_ (V_SCE_)
0	3.18·10^−7^	0.1110	7.2·10^−8^	0.113	7.20·10^−7^	0.0016	6.52·10^−8^	0.0051	12.5·10^−7^	0.0432	1.14·10^−5^	0.401
24	1.41·10^−7^	0.1752	3.56·10^−8^	0.180	3.57·10^−7^	0.1845	2.64·10^−7^	0.191	4.17·10^−7^	0.2271	7.8·10^−9^	0.314
48	0.73·10^−7^	0.1556	1.48·10^−8^	0.199	4.98·10^−7^	0.1377	1.08·10^−7^	0.148	17.28·10^−7^	0.1169	1.23·10^−8^	0.308
72	3.22·10^−7^	0.1257	4.83·10^−7^	0.161	6.24·10^−7^	0.2762	2.51·10^−7^	0.208	19.42·10^−7^	0.2120	6.62·10^−6^	0.400
96	0.56·10^−7^	0.2223	2.72·10^−7^	0.240	3.36·10^−7^	0.2250	1.01·10^−7^	0.234	4.78·10^−7^	0.1914	4.75·10^−6^	0.364

**Table 3:** Parameter values from EIS measurements for AISI 316L at different H_2_O_2_ concentrations and immersion times.

	*R*_pl_ (kΩ cm^2^)	*Q_pl_·*10^−5^ (s^n^ Ω^−1^ cm^−2^)	*n*	*R*_pl_ (kΩ cm^2^)	*Q_pl_·*10^−5^(s^n^ Ω^−1^ cm^−2^)	*n*	*R*_pl_ (kΩ cm^2^)	*Q_pl_·*10^−5^ (s^n^ Ω^−1^ cm^−2^)	*n*
*h*	0.1% H_2_O_2_			5.0% H_2_O_2_			10% H_2_O_2_		
0	9.22	2.57	0.85	26.99	1.78	0.89	26.91	2.15	0.85
24	7.31	4.27	0.90	198.63	1.19	0.88	170.33	1.39	0.92
48	69.51	3.22	0.89	114.96	1.53	0.92	29.78	2.04	0.91
72	65.50	1.97	0.90	28.36	7.37	0.70	21.95	2.76	0.89
96	267.08	2.36	0.86	180.28	1.19	0.81	105.91	1.91	0.90

**Table 4:** Parameter values from EIS measurements for Ti6Al4V at different H_2_O_2_ concentrations and immersion times.

	*R*_pl_ (kΩ cm^2^)	*Q_pl_·*10^−5^ (s^n^ Ω^−1^ cm^−2^)	*n*	*R*_pl_ (kΩ cm^2^)	*Q_pl_·*10^−5^ (s^n^ Ω^−1^ cm^−2^)	*n*	*R*_pl_ (kΩ cm^2^)	*Q_pl_·*10^−5^ (s^n^ Ω^−1^ cm^−2^)	*n*
*h*	0.1% H_2_O_2_			5.0% H_2_O_2_			10.0% H_2_O_2_		
0	86.00	3.40	0.85	6.52	3.25	0.91	8.60	3.02	0.90
24	184.81	3.19	0.94	32.8	4.17	0.88	16.34	24.1	0.86
48	268.00	3.43	0.91	12.39	18.33	0.88	5.19	22.75	0.88
72	89.91	4.64	0.88	6.62	36.05	0.80	4.90	47.54	0.87
96	400.58	4.13	0.93	40.00	4.13	0.94	22.70	3.65	0.68

**Table 5:** Corrosion rates in mm/year for Ti6Al4V and AISI 316L at different H_2_O_2_ concentrations with and time of immersion.

*r* (mm/year)						
	0.1% H_2_O_2_		5.0% H_2_O_2_		10.0% H_2_O_2_	
*h*	Ti6Al4V	AISI 316L	Ti6Al4V	AISI 316L	Ti6Al4V	AISI 316L
0	2.82·10^−3^	7.23·10^−3^	6.23·10^−3^	4.74·10^−3^	11.10·10^−3^	4.82·10^−3^
24	1.25·10^−3^	4.30·10^−3^	3.12·10^−3^	1.54·10^−3^	3.69·10^−3^	2.02·10^−3^
48	0.65·10^−3^	3.04·10^−3^	4.32·10^−3^	1.76·10^−3^	11.12·10^−3^	2.14·10^−3^
72	0.78·10^−3^	2.57·10^−3^	5.41·10^−3^	4.69·10^−3^	12.05·10^−3^	9.01·10^−3^
96	0.49·10^−3^	2.17·10^−3^	2.91·10^−3^	1.57·10^−3^	3.08·10^−3^	2.52·10^−3^

## Data Availability

The original contributions presented in the study are included in the article, further inquiries can be directed to the corresponding author.
